# *In vitro* prediction of clinical signs of respiratory toxicity in rats following inhalation exposure

**DOI:** 10.1016/j.crtox.2021.05.002

**Published:** 2021-05-21

**Authors:** E. Da Silva, C. Hickey, G. Ellis, K.S. Hougaard, J.B. Sørli

**Affiliations:** a Technical University of Denmark, Kgs. Lyngby, Denmark; b National Research Centre for the Working Environment, Copenhagen, Denmark; c Firmenich Incorporated, United States; d Firmenich SA, Switzerland; e University of Copenhagen, Copenhagen, Denmark

**Keywords:** Inhalation toxicity, Lung surfactant, Validation, *In vitro*, OECD test guidelines

## Abstract

To date there are no OECD validated alternative approaches to study toxicity following inhalation exposure to airborne chemicals. The available OECD test guidelines for acute inhalation toxicity aim to estimate a value of the lethal air concentration of the test chemical leading to the death of 50% of the exposed animals (LC_50_), to satisfy hazard classification and labelling requirements. This paper explores the view that alternative approaches must compare to outcomes of existing guideline methods to become accepted and implemented in a regulatory context. This case study describes the initiatives taken to validate the lung surfactant bioassay, an *in vitro* cell-free method, and discusses the challenges faced. While the lung surfactant bioassay could not predict the GHS classification for acute inhalation toxicity of 26 chemicals, the assay successfully predicted the clinical signs of respiratory toxicity observed during or shortly after exposure *in vivo* as reported in registration dossiers. The lung surfactant bioassay is a promising alternative approach to assess the potential of chemicals to cause changes to respiration remaining after exposure (indicating decreased lung function), and can be combined with other test methods in an integrated approach to testing and assessment of inhaled substances.

## Highlights

• OECD TGs for acute inhalation toxicity testing rely on animal studies.
• OECD TGs for acute inhalation toxicity testing focus on systemic effects (LC_50_).
• The *in vitro* lung surfactant bioassay correlates with decreased lung function.
• This bioassay predicts effects caused by lung surfactant function inhibition.

## Graphical abstract

**Figure:**
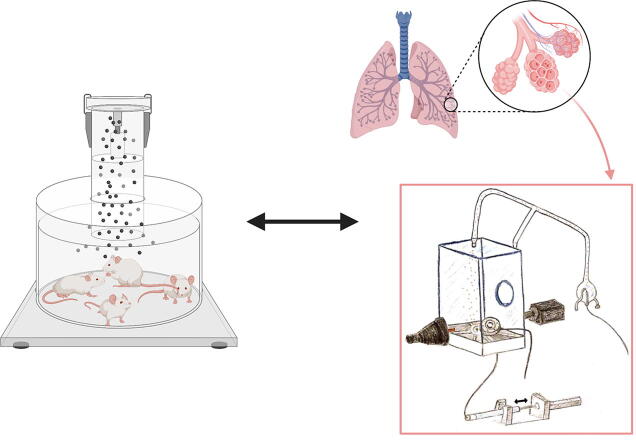

Identification of hazardous properties of airborne chemicals is required under regulatory schemes worldwide, for instance the European regulation for the Registration, Evaluation, Authorisation and Restriction of Chemicals (REACH) (European Commission, 2006), the American Toxic Substances Control Act (TSCA) (Senate and House of Representatives of the United States of America in Congress, 2016), and the South Korean acts on Registration and Evaluation of Chemical Substances and on Consumer Chemical Products and Biocides Safety (The Republic of Korea, 2019, The Republic of Korea, 2020). For regulatory acceptance of test results, internationally accepted testing guidelines (TGs), such as the test methods endorsed by the Organisation for Economic Co-operation and Development (OECD), must be followed (Klimisch et al., 1997). These TGs require acute inhalation toxicity to be assessed in experimental animals and focus on frank toxicity as the endpoint of concern (i.e. evident clinical signs and death). Specifically, the available OECD TGs aim to determine a point or range estimate of the LC_50_, the lethal air concentration of the test chemical leading to the death of 50% of the exposed animals during the observation period, to satisfy hazard classification and labelling requirements of chemicals and mixtures under the United Nations (UN) Globally Harmonized System of Classification and Labelling of Chemicals (GHS) (European Commission, 2008).

According to the OECD TG 403 “Acute Inhalation Toxicity”, a group of rodents is exposed via inhalation to the test substance for up to 4 h and death is the endpoint (OECD, 2009). This guideline was adopted for regulatory use in 1981 but was revised in 2009 to increase flexibility and reduce the number of animals used. The OECD TG 436 “Acute Toxic Class Method”, adopted in 2009, is a sequential procedure, where three animals of each sex are exposed at any of the defined concentration steps to rank substance toxicity (OECD, 2009). It requires fewer animals than the TG 403 but also uses death as the endpoint. The newest OECD TG 433 “Fixed Concentration Procedure” was adopted in 2017 (OECD, 2018). It uses evident clinical signs of toxicity as the endpoint instead of death (OECD, 2018, OECD, 2002). Based on the analysis of a large number of acute inhalation toxicity studies, it was shown that when at least one animal displays evident clinical signs of toxicity (tremors, hypoactivity, irregular respiration, or bodyweight loss), either severe pain and enduring signs of severe distress, moribund condition or mortality would be observed in the majority of the animals at the next highest fixed concentration (Sewell et al., 2015). It is however, questionable whether these OECD TGs and the determination of lethal concentrations are the most human relevant, cost-effective, and ethically sound approaches to discern adverse lung effects following inhalation exposure to chemicals (Da Silva and Sørli, 2018).

REACH regulation states that “animal testing is only acceptable as a last resort, if no other method can be used to provide the necessary information” (European Commission, 2006) and, the Directive 2010/63/EU promotes the development and use of alternative approaches to animal testing with the aim of fully replacing the use of animals in scientific procedures and for educational purposes (European Commission, 2010). In the United States (US), similar efforts to encourage the shift towards non-animal testing have been established in the Frank R. Lautenberg Chemical Safety for the 21st Century Act (Senate and House of Representatives of the United States of America in Congress, 2016) and in the Directive to Prioritize Efforts to Reduce Animal Testing of the US Environmental Protection Agency (EPA, 2019). These acts and directives prompted research within this field, and a growing body of literature describes the efficiency and predictivity of *in silico* and *in vitro* methods to study the toxicity of inhaled chemicals. Recently, a new approach methodology was proposed to the US Environmental Protection Agency by Syngenta Crop Protection for the assessment of inhalation toxicology of chlorothalonil, a respiratory irritant. The assessment combined human relevant simulations and *in vitro* models to derive a point of departure and to calculate human equivalent concentrations (EPA, 2018). Still, there are no validated alternative approaches for hazard identification of inhaled chemicals: the OECD TGs described above remain the basis for regulatory decision-making.

This paper aims to explore the view that alternative approaches must compare to existing methods (OECD TGs) to become accepted and implemented in a regulatory context for hazard identification of chemicals. This is done via a case-study describing the attempts made for the validation of the lung surfactant bioassay, an *in vitro* cell-free method addressing the alveolar region, for hazard identification of airborne compounds and discussing the challenges faced. Twenty-six chemicals were tested *in vitro* in the lung surfactant bioassay, and the test results were compared to the chemicals’ GHS classification for acute inhalation toxicity (based on TG animal studies), and the clinical signs of respiratory toxicity reported within 2 h following inhalation exposure of rats. Lastly, the implementation of the lung surfactant bioassay within an integrated testing strategy for hazard identification of inhaled chemicals is discussed.

## Acceptation and implementation of alternative approaches in a regulatory context

The concept of toxicity pathways was proposed in 2007 (Ankley et al., 2010), and a few years later the OECD programme on development of adverse outcome pathways (AOPs) was initiated (OECD, 2012). AOPs outline the series of steps linking a molecular initiating event to an adverse outcome. It is recognized that understanding the mechanisms underlying toxicity will facilitate the identification of alternative methods targeting specific key events to accelerate the transition away from hazard identification of chemicals based on toxicity testing in animals (Krewski et al., 2010, OECD, 2017).

The validation of alternative methods is currently coordinated by the European Centre for the Validation of Alternative Methods (ECVAM) in Europe and by the US Interagency Coordinating Committee on the Validation of Alternative Methods (ICCVAM) in the United States. The validation process is an essential step of method acceptance. It addresses the purpose of the test method and its mechanistic basis, and assesses its relevance, reliability, and reproducibility (within and among laboratories) (OECD, 2005). This validation is crucial to demonstrate the legitimacy of and increase confidence in alternative approaches. However, the process requires significant investment of time and funding, and the lack of standardisation and harmonisation of data requirements and procedures for validation worldwide hinder the regulatory acceptance and implementation of alternative approaches. A number of barriers to overcome have been discussed in meetings internationally, including the need to make available high-quality reference data, to train on use and interpretation of alternative approaches, and to define a strategy to assess the predictivity and reproducibility of testing strategies in a regulatory context (Table 1 in Supplementary Information). Lastly, the outcomes of the alternative approaches should be “at least as useful as, and preferably better than, the existing method” (cf. OECD Guidance Document 34) (OECD, 2005). It is in itself a challenge to estimate an LC_50_ in rodents (Sperling and McLaughlin, 1976, Zbinden and Flury-Roversi, 1981), so comparison to the outcome of a mechanistically anchored *in vitro* method, with the aim of a correlation, seems hardly possible.

## Predictability of the lung surfactant bioassay for GHS classification of inhaled chemicals

The lung surfactant has a fundamental role in ensuring effortless breathing by decreasing the surface tension at the air–liquid interface in the alveolar regions of the lungs. Lung surfactant lines the inside of the alveoli at the air–liquid interface, and it constitutes the first entity that inhaled chemicals encounter in the alveolar region. Lung surfactant is a complex biological liquid, made of 90% lipids and 10% proteins (including the four surfactant proteins SP-A, SP-B, SP-C and SP-D) (Goerke, 1998, Creuwels et al., 1997).

Interaction of airborne chemicals with lung surfactant in the alveolar region may signify the onset of clinical signs of respiratory toxicity. An example of this is the observation of an extremely steep concentration–response curve in a mouse inhalation model (dramatic and rapid reduction in the tidal volume leaving animals in a moribund state) after exposure to impregnation spray products (Nørgaard et al., 2010, Larsen et al., 2014). It was hypothesized that the effect was driven by a physical interaction of the compound with the lung surfactant rather than the chemical activation of trigeminal or vagal nerve receptors as previously observed for airway irritants (Larsen et al., 2016, Larsen et al., 2009, Nørgaard et al., 2014, Duch et al., 2014). A series of studies in recent years have contributed to the understanding of the mechanism of toxicity of inhaled chemicals and particles and have investigated the predictivity of the lung surfactant bioassay for changes in breathing patterns of mice following inhalation exposure as an indicator of serious toxicity to the lungs. Inhalation exposure to impregnation spray products (Nørgaard et al., 2014, Duch et al., 2014, Sørli et al., 2015, Sørli et al., 2017), bile salts (Sørli et al., 2018), and zinc oxide nanoparticles (Larsen et al., 2020) led to a sudden decrease in tidal volume *in vivo.* This correlated well with inhibition of lung surfactant function *in vitro* in the lung surfactant bioassay. At the molecular level, investigations of chemically-induced inhibition of the lung surfactant function identified that the test chemicals intercalated between the surfactant phospholipids at the air–liquid interface, reduced the stability of the lung surfactant films, and induced the loss of cohesivity of the multi-layered surfactant structures as the main perturbations (Da Silva et al., 2021b). At the organism level in humans, impregnation spray products that caused adverse respiratory outcomes after incidental exposure were also inhibitory to the lung surfactant function *in vitro* (Duch et al., 2014, Sørli et al., 2017). In addition, inhibition of the lung surfactant function is involved in several respiratory disorders in humans, including acute respiratory distress syndrome (Touqui and Arbibe, 1999, Gunther et al., 2001, Autilio and Perez-Gil, 2019, Echaide et al., 2017). Based on these findings, the AOP 302 was proposed (Halappanavar et al., 2020; Da Silva et al., 2021a) and www.aopwiki.org/aops/302). It describes the series of events starting from the interaction of chemicals with the lung surfactant that lead to decreased lung function (Fig. 1 in Supplementary Information). The weight of evidence supporting this AOP and a description of the methods that can be used to asses each key event are described in an accompanying paper in this journal (Da Silva et al., 2021a).

These observations gave rise to the idea that the lung surfactant bioassay could be used for hazard identification of airborne chemicals and in early product development. It became a stated ambition that this alternative approach could – in the context of an integrated approach to testing and assessment – replace *in vivo* experiments for regulatory testing of inhaled chemicals (Da Silva and Sørli, 2018). Therefore, it was our hypothesis that the lung surfactant bioassay could predict the GHS classification of chemicals obtained according to the OECD TGs 403 and 436 for acute inhalation toxicity.

## Methods

Twenty-six chemicals were selected for investigation of their potential for inhibition of lung surfactant function, the CAS numbers and chemical formulas of all chemicals can be found in Table 2 in Supplementary Information. All chemicals had been tested previously according to the OECD TGs 403 and 436 due to regulatory requirements the LC_50_ (mg/L) and classification can be found in Table 2 in Supplementary Information. The first subgroup included nine fragrance materials that were tested for acute inhalation toxicity as a regulatory requirement to enter the market (supplied by Firmenich Incorporated, United States). The second subgroup contained 17 chemicals registered under REACH and selected from the eChemPortal platform using the property search function (*Toxicological information* > *Acute Toxicity* > *Acute toxicity: inhalation*) (eChemPortal, 2017). Criteria for the selection of chemicals were: study “reliable without restriction”, animals exposed “nose only”, “head only” or “nose/head only”, and chemical classification under category 3, category 4, or “conclusive but not sufficient for classification”. Using these selection criteria, a total of 353 chemicals were identified (search performed in December 2017). From these, 17 chemicals were selected based on: their relevance for workers' and consumers' exposure (by reading the “Manufacture, use & exposure” section in the registration dossier) and the presence of an apparent dose–response relationship described in the registration dossier (based on the number of deaths at each concentration level). Based on these criteria, six chemicals without classification, and nine chemicals with a classification were selected. These 17 chemicals were acquired at the highest available purity from Sigma-Aldrich, Denmark. Three of the chemicals were further studied to determine the mechanisms underlying lung surfactant function inhibition, for further details see Da Silva et al 2021 (Da Silva et al., 2021b). Information relating to the TGs outcomes (both LC_50_ and clinical observations) were provided by Firmenich or extracted from the available REACH registration dossiers. The clinical signs of respiratory toxicity observed during and within two hours post-exposure are indicated in Table 2 in Supplementary Information.

The lung surfactant function was evaluated *in vitro* in the constrained drop surfactometer (BioSurface Instruments, United States, (Valle et al., 2015) as previously described in detail (Sørli et al., 2017). Briefly, a drop of surfactant (10 µL) was placed on a knife-edge pedestal and underwent cyclic compressions and expansions imposed by a syringe pump to mimic human respiratory cycles (20 cycles/min). The effects of the fragrance materials on lung surfactant function were assessed using Curosurf® (Chiesi, Italy, 2.5 mg/mL), while the chemicals identified from eChemPortal were tested using purified native porcine surfactant (1.0 mg/mL). The surface activity of the two surfactant models was comparable (Fig. 1 in Supplementary Information). In both cases, the diluting buffer was made of 0.9% NaCl, 1.5 mM CaCl_2_, and 2.5 mM HEPES, and adjusted to pH 7.0, and the dilutions were made in the morning of each day of experiments.

Liquid chemicals were used undiluted, and chemicals in powder form were dissolved in deionized water near their limit for water solubility. The substances were blinded for the operator using an assigned letter for identification. All tested chemicals were led from a glass syringe into a Pitt no.1 jet nebulizer (Wong and Alarie, 1982) by means of a syringe pump (Legato 100, Buch & Holm A/S, Denmark) to the exposure chamber and deposited onto the drop of lung surfactant. Although particle size distribution in the generated aerosols was not measured in this study, there is empirical evidence that the particles generated with the Pitt no. 1 jet nebulizer have an aerodynamic parameter below 5 µm, i.e. in the respirable range (Sørli et al., 2015, Sørli et al., 2018, Larsen et al., 2020, Da Silva et al., 2021b). To ensure continuous air flow through the exposure chamber, the air was sucked out through the ventilation holes in the hollow baseplate. The pressurized air and exposure chamber were heated to 37 °C, and the temperature inside the exposure chamber was monitored using the TinyTag Plus 2 data logger (TGP-4017, Gemini Data Loggers Ltd, United Kingdom). The drop of lung surfactant was exposed to the test chemical, and images of the drop were captured throughout the experiment (10 frames per second) and analysed using the axisymmetric drop shape analysis (ADSA) (Yu et al., 2016) to yield surface tension values. Each experiment was repeated three to five times.

For each compression-expansion cycle, the values of the minimum surface tension (reached at the minimum surface area of the drop) were extracted. The minimum surface tension of the baseline should be below 5 mN/m with a compression (decrease in surface area) below 30% for acceptance of data. The inhibition of the lung surfactant function was defined as an increase in the minimum surface tension to values greater than or equal to 10 mN/m for three or more consecutive minima. *In vivo*, an increase of the minimum surface tension beyond 10 mN/m will lead to alveolar collapse (Enhorning, 2001).

### Identification of chemicals classified under GHS for acute inhalation toxicity

Based on the criteria listed above, each of the tested chemicals were defined as either inhibitory to the lung surfactant function or not. Test chemicals falling under GHS categories 3 and 4 for acute inhalation toxicity were identified as “classified”. A compound which was found to inhibit the lung surfactant function and was classified as an acute inhalation toxicant under GHS, was considered as a true positive. Based on the binary outcomes of these independent *in vitro* and *in vivo* tests, the following predictive accuracy metrics were calculated: (*i*) sensitivity (the ability to detect a true positive) and specificity (the ability to detect a true negative), indicating the concordance between the lung surfactant bioassay outcome and the GHS classification for acute inhalation toxicity, (*ii*) positive and negative predictive values, as indication of the likelihood that the bioassay can successfully identify the hazard classification, and (*iii*) accuracy (Table 3 in Supplementary Information) (Trevethan, 2017).

The analysis shows that across all metrics tested, the lung surfactant bioassay results correlated poorly with the GHS classifications for acute inhalation toxicity: the assessments were accurate for only 50% of the chemicals (Table 1).

**Table 1:** Predictivity metrics for the lung surfactant bioassay for the two scenarios.

	GHS classification	Clinical signs of respiratory toxicity
Sensitivity	0.67 (10/15)	0.81 (17/21)
Specificity	0.27 (3/11)	0.80 (4/5)
Positive predictive value	0.56 (10/18)	0.94 (17/18)
Negative predictive value	0.38 (3/8)	0.50 (4/8)
Accuracy	0.50 (13/26)	0.81 (21/26)

### Predicting clinical signs of respiratory toxicity in rats

The lung surfactant bioassay addresses a local (alveolar region) and immediate effect, occurring within a few minutes from the start of the exposure. For this reason, the *in vitro* results were compared to the clinical signs of respiratory toxicity observed in rats during exposure (H0) and up to 2 h (H1 and H2) post-exposure, to represent decreased lung function in the rat. The clinical observations reported in the dossiers were assigned a standard clinical sign (irregular-, shallow-, noisy-, slow-, rapid/fast-respiration, and gasping) based on the lexicon developed by Sewell and co-authors (Sewell et al., 2015) (Table 4 in Supplementary Information). The predictive accuracy metrics were re-defined as follows: (*i*) the sensitivity and the specificity indicated concordance of the lung surfactant bioassay to the clinical signs of respiratory toxicity *in vivo*, (*ii*) the positive and negative predictive values indicated the likelihood that the lung surfactant bioassay successfully identifies the chemicals that caused clinical signs of respiratory toxicity *in vivo* up to 2 h post-exposure.

This analysis shows that the lung surfactant bioassay correctly identified 81% of the chemicals that caused clinical signs of respiratory toxicity in rats (sensitivity) and 80% of the chemicals that did not (specificity). The positive and negative predictive values were 94% and 50%, respectively (Table 1).

## Discussion

From a precautionary perspective, the ratio of false negative outcomes must be low (for the protection of human health), whereas a higher ratio of false positives will be more acceptable although it may generate unnecessary risk management measures (Griesinger, 2016). With an overall accuracy of 81%, the performance of the lung surfactant assay to predict clinical signs of respiratory toxicity was far superior to that of the prediction of the GHS classification for acute inhalation toxicity.

Several reasons can be identified for the lack of correlation between the outcomes of the lung surfactant bioassay and the GHS classification of the chemicals. First and foremost, the compared endpoints are fundamentally different. While the current OECD TGs assess systemic toxicity by estimating the LC_50_ value after inhalation in rodents, the lung surfactant bioassay is anchored in the biological pathway leading to decreased lung function due to local effects in the alveoli. There could be numerous causes of death of the rodents, which may, or may not, be related to adverse effects in the lungs. Other reasons for the poor predictivity metrics include the use of the LC_50_ value as basis for classification and the differences in the chronology of the endpoints investigated *in vitro* and *in vivo*. Low prediction accuracy of *in vitro* models for acute toxicity is not new: the ACUTox Project investigated a number of *in vitro* methods alone and in combination and studied their predictive capacity for the GHS classes of acute oral toxicity. The results led the researchers “to question the scientific motivation for the currently applied classification systems that are based on arbitrary cut-off values for rat oral LD_50_ values used to estimate human acute oral toxicity and to further advise a revision of the current classification system” (Prieto et al., 2013, Hoffmann et al., 2010). The findings of the present study support the recently outlined challenge of the use of arbitrary LC_50_ ranges, which are not related to specific mechanisms, for classification of acute toxicity into hazard categories (Prieto, 2019). Interestingly, the OECD TGs for acute inhalation toxicity state that “when possible, any differentiation between local and systemic effects should be noted.” However, in practice, this information is rarely available in the REACH registration dossiers and, if present, it does not address the type and severity of the lesions, their persistence or reversibility, nor the mechanism of toxicity of the inhaled test chemicals.

### Towards an integrated approach to testing and assessment of inhaled chemicals

The inhibition of lung surfactant function by chemicals, that is evaluated by the lung surfactant bioassay, represents only one key event in the pathway leading to decreased lung function. The lung surfactant bioassay cannot predict the adverse effects of airborne chemicals that occur via lung surfactant-independent mechanisms. For that reason, the lung surfactant bioassay is not sufficient as a stand-alone method to conclude on the potential for decreased lung function and it should be combined with other methods within an integrated approach to testing and assessment of airborne chemicals.

To start with, it is of primary importance to consider the physico-chemical properties of the test chemicals because this governs: the exposure, the target region in the lungs, the choice of a suitable test methods (according to the applicability domain), and the waiving of specific endpoints (OECD, 2016). Among other parameters, the biosolubility of a test chemical is important to understand its dissolution behaviour in the lungs. This parameter was recently used by the US Environmental Protection Agency to revoke the significant new use rule (SNUR) in 2020 under the Toxic Substances Control Act (TSCA): based on the solubility of alpha 1,3-polysaccharide in simulated epithelial lung fluid. It was concluded that the compound could not be considered poorly soluble and that the associated hazard concern for lung overload was low (EPA, 2020). We refer to (Clippinger et al., 2018) for a review of the physico-chemical parameters of interest in the context of inhalation toxicity testing (Clippinger et al., 2018). Thereafter, relevant complementary assays for the identification of the potential for adverse lung effects can be considered. These include non-testing methods (*in silico*, read-across from chemical analogues), and *in vitro* methods exploring: inflammatory response (release of pro-inflammatory cytokines and chemokines at gene, protein, and inflammasome level); generation of reactive oxygen species; disruption of the membrane integrity; cytotoxicity; ciliary beating frequency; mucus production; and genotoxicity (DNA strand breaks; mutation frequencies; genome instability; chromosome aberration). Combining several alternative approaches also has the advantage of covering a larger range of chemicals, because the applicability domain of each method may not overlap.

In the broader context of validation and regulatory acceptance of alternative approaches, assessment of skin sensitization is ahead of other health effects. Here, the steps of the adverse outcome pathway are well understood (the AOP for skin sensitization was published by the OECD in 2012, (OECD, The Adverse Outcome Pathway for Skin Sensitisation Initiated by Covalent Binding to Proteins. OECD Series on Testing and Assessment, No. 168, 2014), and alternative approaches are available to investigate the specific key events. Since 2016, REACH regulation requires the use of *in vitro* and *in silico* methods as a first choice for this endpoint. On these grounds, the lessons learned for the transition from the local lymph node assay (LLNA) *in vivo* to the current integrated approach of testing and assessment provide valuable directions for the future of replacement in the context of inhalation toxicity testing.

One of the available assays for skin sensitization is the Direct Peptide Reactivity Assay (DPRA). During the validation study against the LLNA assay, the accuracy was 80% (n = 157), the sensitivity was 80% (88/109), and the specificity was 77% (37/48) (OECD, 2020). Similarly, to the DPRA addressing the covalent binding of test chemicals to proteins *in chemico,* the lung surfactant bioassay relies on the identification of a physical interaction, of the chemical with the lung surfactant in the alveolar region. It is encouraging that the predictivity of the lung surfactant bioassay for clinical signs of respiratory toxicity *in vivo* compares well with the predictivity of this regulatory accepted and implemented test method.

## Methodological considerations

The findings reported here might be somewhat limited by the following aspects: the sample size in the current study is small; the predictivity values were calculated for the lung surfactant bioassay as a stand-alone method, whereas in an integrated approach to testing and assessment of airborne chemicals it would be combined with other assays; the aim of the TG studies was not to identify clinical signs of respiratory toxicity but to estimate LC_50_; and outcomes of the TG studies in rats may not reflect effects following exposure in humans. Also, a note of caution is due regarding lack of precision in the reported respiratory outcomes in exposed rats: the description of changes in breathing patterns relies on cage-side visual observations, and the number of occurrences in the group of exposed animals varies (some signs were experienced by one animal, others by the entire group). Furthermore the reported clinical signs of respiratory toxicity could be due to other mechanisms than those triggered by disruption of lung surfactant function, e.g. by inducing reflexes by stimulating different nerve endings in the lungs (Nielsen et al., 2005).

The physico-chemical properties of the test chemicals may affect the measurement of the inhibitory dose in the lung surfactant bioassay. In this *in vitro* system, a quartz crystal microbalance (QCM) placed close to the drop of lung surfactant in the exposure chamber measures the deposited mass of the test chemical in real time in ng/cm^2^. Knowing both the average area of the cycling lung surfactant drop, and the time at inhibition, the inhibitory dose of the test chemical can be estimated. These measurements are reliable for non- to low volatile compounds and have been used to extrapolate *in vitro* inhibitory doses to *in vivo* exposure scenarios previously (Sørli et al., 2018, Sørli et al., 2015, Larsen et al., 2020). Nevertheless, certain physico-chemical characteristics of the test chemicals may prevent such calculations. This is the case for highly volatile compounds, such as the nine fragrance materials included in this study, for which the measurement of deposited mass in the exposure chamber (with its high temperature and high air flow rate) over time was unreliable.

In addition to affecting the assessment of dosimetry in the lung surfactant bioassay, the high volatility of certain compounds, and their fugacity capacity, can dictate their interaction with the lung surfactant. Other characteristics such as water solubility, boiling point (if very low) and hydrophobicity have been proposed to rule the molecular and biophysical disruption of lung surfactant function (Da Silva et al., 2021b). These parameters were not collected in the current study, but the chemical structure (Table 2 in Supplementary Information) indicates that they vary across the 26 test chemicals. Understanding the correlation between specific physico-chemical properties and the disruption of lung surfactant function will be an important point to investigate in the future.

The systematic definition of methods employed to record clinical signs of toxicity, and the improvement of the precision of the results reported in the REACH registration dossiers are necessary in order to broaden their use by the scientific community (Ågerstrand et al., 2018). The quantitative translation to human exposure scenarios is not possible in the present study. As mentioned previously, it was not possible to estimate the inhibitory dose for many of the chemicals because of high volatility. This limitation will have to be overcome before translation to human exposure can be done.

## Conclusion

There are no validated alternative approaches for hazard identification of inhaled chemicals. The OECD TGs, relying on severe systemic effects in experimental animals, remain the basis for regulatory decision-making. This is in spite of the ongoing global effort to investigate and predict respiratory outcomes of inhaled substances *in vitro* and *in silico*, and the recognized need to develop AOPs. Beyond the scientific challenges, this paper discusses the current perception that outcomes of alternative approaches must correlate to standardized endpoints (like the LC_50_) of existing methods to become accepted in the regulatory context and implemented for the hazard identification of chemicals. This concept ignores the possibility that mechanistic alternative approaches may be more relevant for assessment of hazards to human health, such as in this case of inhalation toxicity testing. This study found that the lung surfactant bioassay, anchored in a biological pathway, could not predict the GHS classification for acute inhalation toxicity of 26 chemicals; however, the assay did show good correlation to reported clinical signs of respiratory toxicity *in vivo*. Combined with other test methods in an integrated approach to testing and assessment, the lung surfactant bioassay can provide invaluable information to study clinical signs of respiratory toxicity which are not adequately covered by current animal testing procedures and outcomes.

## CRediT authorship contribution statement

**E. Da Silva:** Conceptualization, Investigation, Formal analysis, Writing - original draft. **C. Hickey:** Resources, Writing - review & editing. **G. Ellis:** Resources, Writing - review & editing. **K.S. Hougaard:** Writing - review & editing, Supervision. **J.B. Sørli:** Conceptualization, Methodology, Writing - review & editing.

## Declaration of Competing Interest

The authors declare the following financial interests/personal relationships which may be considered as potential competing interests: Christina Hickey is an employee of Firmenich Inc., a company supplying fragrance and flavours for use in consumer products. Graham Ellis is an employee of Firmenich SA, a company supplying fragrance and flavours for use in consumer products.
