# Supplementary material for: In vitro prediction of clinical signs of respiratory toxicity in rats following inhalation exposure
Source: Curr Res Toxicol. 2021 May 21;2:204–9. doi: 10.1016/j.crtox.2021.05.002 (PMC8320621; doi:10.1016/j.crtox.2021.05.002)
Supplement: Supplementary Data 1 [file mmc1.docx]

**Supplementary information**

**Supplementary information**


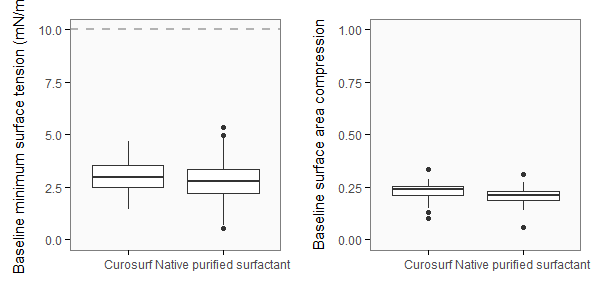


Figure 1: comparison of the surface activity of the two surfactant models Curosurf and native purified surfactant. Left: mean of the minimum surface tension. Right: mean of the compressibility.

Table 1: Overview of the initiatives taken and peer-reviewed articles published to address acute systemic and acute inhalation toxicity internationally

| **Workshop title** |  | **Organizer** | **Year** |
| --- | --- | --- | --- |
| Alternative approaches for identifying acute systemic toxicity: moving from research to regulatory testing | (Hamm, Sullivan et al. 2017) |  | 2015 |
| Strategy for the ultimate elimination of the acute toxicity “six-pack”  -update on OPP progress  -letter to stakeholders | (2016)  (2016) | US EPA OPP | 2016 |
| Evaluation of a proposed approach to refine the inhalation risk assessment for point of contact toxicity: a case study using a new approach methodology  -document to the FIFRA scientific advisory panel  -scientific advisory panel report | (2018)  (2018) | US EPA | 2018 |
| Alternative approaches for acute inhalation toxicity testing to address global regulatory and non-regulatory data requirements  -workshop  -webinar series | (Clippinger, Allen et al. 2018)  (PETA-ISC 2016) | NICEATM and PETA-ISC | 2016 |
| Toxicology showcase event | (Sewell, Doe et al. 2017) | NC3Rs | 2016 |
| Validation of alternative methods | (Piersma, Burgdorf et al. 2018) | BfR-RIVM | 2017 |
| Non-animal approaches for inhalation toxicity testing | (ICCVAM 2019) | ICCVAM | 2019 |
| **Peer-reviewed articles (Schiffelers, Blaauboer et al. 2014, Burden, Sewell et al. 2015, Prior, Casey et al. 2019)** |  |  |  |
|  |  |  |  |

*OPP: Office of Pesticide Programs; US EPA: United States Environmental Protection Agency; FIFRA: Federal Insecticide, Fungicide, and Rodenticide Act; NICEATM: National Toxicology Program Interagency Center for the Evaluation of Alternative Toxicological Methods; PETA-ISC: People for the Ethical Treatment of Animals – International Science Consortium; NC3Rs: National Centre for the Replacement Refinement & Reduction of Animals in Research; BfR: German Federal Institute for Risk Assessment; RIVM: Dutch National Institute for Public Health and the Environment; ICCVAM: Interagency Coordinating Committee on the Validation of Alternative Methods.*

Table 2: definition of sensitivity, specificity, positive predictive value, negative predictive value, accuracy

|  | **Positive result *in vivo*** | **Negative result *in vivo*** |
| --- | --- | --- |
| **Positive result *in vitro*** | (A) True positive | (B) False positive |
| **Negative result *in vitro*** | (C) False negative | (D) True negative |

Sensitivity = A / (A+C) ; Specificity = D / (B+D)

Positive predictive value = A / (A+B) ; Negative predictive value = D / (C+D)

Accuracy = (A+D) / (A+B+C+D)

Figure 2: Diagram illustrating the adverse outcome pathway 302 (AOP). The AOP starts with the inhibition of lung surfactant function that is responsible for a series of key events including alveolar collapse, reduced tidal volume, and loss of capillary membrane integrity. These key events lead to impaired lung function that is characterized by respiratory clinical signs of toxicity and reduced oxygenation of the blood.

Table 3: CAS number, chemical name and structure, LC_50_, GHS classification for acute inhalation toxicity, clinical signs of respiratory toxicity observed in rats following inhalation exposure, and inhibition *in vitro* in the lung surfactant bioassay.

| **CAS** | **Chemical name** | **Structure** | **LC_50_ (mg/L)** | **GHS classification** | **Clinical signs (time of onset)** | **Inhibition *in vitro*** |
| --- | --- | --- | --- | --- | --- | --- |
| 546-80-5 | (−)-α-Thujone |  | <5 | Acute Tox. 3 | - | Yes |
| 774-55-0 | 6-Acetyl-1,2,3,4-tetrahydronaphthalene |  | >5 | N/A | Slow respiration (H1) | Yes |
| 79893-63-3 | 6-ethyl-2,10,10-trimethyl-1-oxaspiro[4.5]deca-3,6-diene |  | >5 | N/A | Laboured respiration/dyspnea (H1) | Yes |
| 2206-94-2 | alpha-methylenebenzyl acetate |  | >5 | N/A | Slow respiration (H1) | Yes |
| 35087-49-1 | 2-Buten-1-one,1-(2,2-dimethyl-6-methylenecyclohexyl) |  | 3.5 | Acute Tox. 4 | Noisy respiration (H1) Slow respiration (H1) | Yes |
| 7774-82-5 | 2-Tridecenal |  | 1<LC50<5 | Acute Tox. 4 | Irregular respiration (H1) Laboured respiration/dyspnea (H1) Noisy respiration (H1) | Yes |
| 130786-09-3 | Benzeneacetonitrile, alpha-butylidene-, (alphaZ)- |  | >5 | Acute Tox. 4 for females | Slow respiration (H1) | Yes |
| 120811-92-9 | Benzene,(3-methoxy-2-methylpropyl)- |  | >5 | N/A | Slow respiration (H1) | Yes |
| 143-14-6 | Benzene,(3-methoxy-2-methylpropyl)- |  | >5 | Acute Tox. 4 | Laboured respiration/dyspnea (H1) Slow respiration (H1) | Yes |
| 2182-55-0 | (vinyloxy)cyclohexane |  | >22.007 | conclusive but not sufficient for classification (OECD TG 403) | Slow respiration (H1) Intermittent respiration (H1) Laboured respiration/dyspnea (H1) | Yes |
| 544-01-4 | 1,1'-oxybis(3-methylbutane) |  | 1.9-7.1 | Acute Tox. 3 (OECD TG 436) | Slow respiration (H0) | Yes* |
| 133-06-2 | 1,2,3,6-tetrahydro-N-(trichloromethylthio)phthalimide |  | 0.78 | Acute Tox. 3 (OECD TG 403) | Slow respiration (H0) Gasping (H1) | No |
| 1477-55-0 | 1,3-bis(aminomethyl)benzene |  | 1.34 | Acute Tox. 4 (OECD TG 403) | Noisy respiration (H0) Rapid/fast respiration (H0) | No |
| 2687-91-4 | 1-ethylpyrrolidin-2-one |  | >5.1 | conclusive but not sufficient for classification (OECD TG 403) | Rapid/fast respiration (H0) | No |
| 541-02-6 | 2,2,4,4,6,6,8,8,10,10-decamethyl-1,3,5,7,9,2,4,6,8,10-pentoxapentasilecane |  | 8.67 | GHS criteria not met (OECD TG 403) | Shallow respiration (H0) | Yes |
| 110-65-6 | 2-butyne-1,4-diol |  | 0.69 | Acute Tox. 3 (OECD TG 403) | - | No |
| 91-76-9 | 6-phenyl-1,3,5-triazine-2,4-diamine |  | 2.932 | Acute Tox. 4 (OECD TG 403) | Shallow respiration (H0) | No |
| 16893-85-9 | disodium hexafluorosilicate |  | 1.81 | Acute Tox. 3 (OECD TG 403) | - | No |
| 140-88-5 | ethyl acrylate |  | <9.137 | Acute Tox. 3 (OECD TG 403) | Noisy respiration (H0) | Yes |
| 24851-98-7 | Methyl 3-oxo-2-pentyl-1-cyclopentaneacetate |  | >4.93 | conclusive but not sufficient for classification (OECD TG 403) | Rapid/fast respiration (H0) | Yes* |
| 96-33-3 | methyl acrylate |  | <10.832 | Acute Tox. 3 (OECD TG 403) | Slow respiration (H2) Gasping (H2) Noisy respiration (H2) | Yes |
| 111-82-0 | methyl laurate |  | >5 | conclusive but not sufficient for classification (OECD TG 436) | Laboured respiration/dyspnea (H1) | Yes |
| 111-81-9 | methyl undec-10-enoate |  | 3.76 | Acute Tox. 4 (OECD TG 403) | Shallow respiration (H0) Irregular respiration (H2) | Yes |
| 7757-79-1 | potassium nitrate |  | >0.527 | conclusive but not sufficient for classification (OECD TG 403) |  | No |
| 79710-86-4 | tetrahydrofuran-3-carbaldehyde |  | >4.48 | conclusive but not sufficient for classification (OECD TG 403) |  | No |
| 3069-40-7 | trimethoxy(octyl)silane |  | 3.9 | Acute Tox. 3 (OECD TG 403) | Irregular respiration (H1) Shallow respiration (H1) | Yes* |

*H0: occurrence during exposure. H1: occurrence during the first hour post-exposure. H2: occurrence during the second hour post-exposure.*

* also studied in (Da Silva, Autilio et al. 2020)

Table 5: Clinical signs reported in the registration dossiers for the test chemicals and their respective standardised version, in agreement with the standardized clinical signs described by (Sewell, Ragan et al. 2015)

| **Clinical sign as reported** | **Standardised clinical sign** |
| --- | --- |
| Breathing patterns were irregular | Irregular respiration |
| Breathing patterns were superficial | Shallow respiration |
| Rales | Noisy respiration |
| Depressed respiration | Slow respiration |
| Intermittent respiration | Irregular respiration |
| Decreased respiratory rate | Slow respiration |
| Frequent sneezing | Sneezing |
| Pulmonary respiration sounds | Noisy respiration |
| Visually accelerated respiration | Rapid/fast respiration |
| Tachypnea | Rapid/fast respiration |
| Panting | Rapid/fast respiration |
| Respiratory murmur | Noisy respiration |
| Respiration sounds | Noisy respiration |
| Increased respiratory rate | Rapid/fast respiration |
| Shallow breathing | Shallow respiration |

Signs present in the lexicon (Sewell, Ragan et al. 2015) to which OECD refers:

-Irregular respiration

-Slow respiration

-Gasping

-Rapid/fast respiration

-Laboured respiration/dyspnea

-Noisy respiration

-Sneezing

-Shallow respiration

References

(2016). Letter to Stakeholders on EPA Office of Pesticide Programs's Goal to Reduce Animal Testing from Jack E. Housenger, Director Office of Pesticide Programs, US Environmental Protection Agency.

(2016). Update on OPP Progress on Acute Animal Testing Alternatives PPDC Meeting Nov. 3, 2016 – Session 7a US Environmental Protection Agency - Office of Pesticides Program.

(2018). "Evaluation of a Proposed Approach to Refine the Inhalation Risk Assessment for Point of Contact Toxicity: A Case Study Using a New Approach Methodology (NAM).", from https://www.epa.gov/pesticides/fifra-sap-meeting-evaluation-proposed-approach-refine-inhalation-risk-assessment-point.

(2018). FIFRA Scientific Advisory Panel Meeting Minutes and Final Report No. 2019-01 Peer Review on Evaluation of a Proposed Approach to Refine the Inhalation Risk Assessment for Point of Contact Toxicity: A Case Study Using a New Approach Methodology (NAM) December 4 and 6, 2018 FIFRA Scientific Advisory Panel Meeting, US Environmental Protection Agency.

Burden, N., F. Sewell and K. Chapman (2015). "Testing Chemical Safety: What Is Needed to Ensure the Widespread Application of Non-animal Approaches?" PLOS Biology **13**(5): e1002156.

Clippinger, A. J., D. Allen, A. M. Jarabek, M. Corvaro, M. Gaça, S. Gehen, J. A. Hotchkiss, G. Patlewicz, J. Melbourne, P. Hinderliter, M. Yoon, D. Huh, A. Lowit, B. Buckley, M. Bartels, K. BéruBé, D. M. Wilson, I. Indans and M. Vinken (2018). "Alternative approaches for acute inhalation toxicity testing to address global regulatory and non-regulatory data requirements: An international workshop report." Toxicology in Vitro **48**: 53-70.

Da Silva, E., C. Autilio, K. S. Hougaard, A. Baun, A. Cruz, J. Perez-Gil and J. B. Sørli (2020). "Molecular and biophysical basis for the disruption of lung surfactant function by chemicals." Biochim Biophys Acta Biomembr **1863**(1): 183499.

Hamm, J., K. Sullivan, A. J. Clippinger, J. Strickland, S. Bell, B. Bhhatarai, B. Blaauboer, W. Casey, D. Dorman, A. Forsby, N. Garcia-Reyero, S. Gehen, R. Graepel, J. Hotchkiss, A. Lowit, J. Matheson, E. Reaves, L. Scarano, C. Sprankle, J. Tunkel, D. Wilson, M. Xia, H. Zhu and D. Allen (2017). "Alternative approaches for identifying acute systemic toxicity: Moving from research to regulatory testing." Toxicology in Vitro **41**: 245-259.

ICCVAM. (2019). "ICCVAM Communities of Practice Webinar 2019 on Non-animal Approaches for Inhalation Toxicity Testing." from https://ntp.niehs.nih.gov/whatwestudy/niceatm/3rs-meetings/past-meetings/commprac-2019/commprac-2019.html.

PETA-ISC, N. a. (2016). "Alternative Approaches for Acute Inhalation Toxicity Testing." from https://www.piscltd.org.uk/acute_inhalation_toxicity/.

Piersma, A. H., T. Burgdorf, K. Louekari, B. Desprez, R. Taalman, R. Landsiedel, J. Barroso, V. Rogiers, C. Eskes, M. Oelgeschläger, M. Whelan, A. Braeuning, A. M. Vinggaard, A. Kienhuis, J. van Benthem and J. Ezendam (2018). "Workshop on acceleration of the validation and regulatory acceptance of alternative methods and implementation of testing strategies." Toxicology in Vitro **50**: 62-74.

Prior, H., W. Casey, I. Kimber, M. Whelan and F. Sewell (2019). "Reflections on the progress towards non-animal methods for acute toxicity testing of chemicals." Regulatory Toxicology and Pharmacology **102**: 30-33.

Schiffelers, M.-J., B. J. Blaauboer, W. E. Bakker, S. Beken, C. F. M. Hendriksen, H. B. W. M. Koëter and C. Krul (2014). "Regulatory acceptance and use of 3R models for pharmaceuticals and chemicals: Expert opinions on the state of affairs and the way forward." Regulatory Toxicology and Pharmacology **69**: 41-48.

Sewell, F., J. Doe, N. Gellatly, I. Ragan and N. Burden (2017). "Steps towards the international regulatory acceptance of non-animal methodology in safety assessment." Regulatory Toxicology and Pharmacology **89**: 50-56.

Sewell, F., I. Ragan, T. Marczylo, B. Anderson, A. Braun, W. Casey, N. Dennison, D. Griffiths, R. Guest, T. Holmes, T. van Huygevoort, I. Indans, T. Kenny, H. Kojima, K. Lee, P. Prieto, P. Smith, J. Smedley, W. S. Stokes, G. Wnorowski and G. Horgan (2015). "A global initiative to refine acute inhalation studies through the use of 'evident toxicity' as an endpoint: Towards adoption of the fixed concentration procedure." Regul Toxicol Pharmacol **73**(3): 770-779.
